# Role of Human Cytomegalovirus Tegument Proteins in Virion Assembly

**DOI:** 10.3390/v6020582

**Published:** 2014-02-06

**Authors:** Rebecca Marie Smith, Srivenkat Kosuri, Julie Anne Kerry

**Affiliations:** Department of Microbiology and Molecular Cell Biology, Eastern Virginia Medical School, Norfolk, VA 23501, USA; E-Mails: beckstermw@yahoo.com (R.S.); kosurisr@evms.edu (S.K.)

**Keywords:** human cytomegalovirus, tegument, assembly

## Abstract

Like other herpesviruses, human cytomegalovirus (HCMV) contains a unique proteinaceous layer between the virion envelope and capsid, termed the tegument. Upon infection, the contents of the tegument layer are delivered to the host cell, along with the capsid and the viral genome, where they facilitate the initial stages of virus replication. The tegument proteins also play important roles in virion assembly and this dual nature makes them attractive potential targets for antiviral therapies. While our knowledge regarding tegument protein function during the initiation of infection has been the subject of intense study, their roles in assembly are much less well understood. In this review, we will focus on recent studies that highlight the functions of HCMV tegument proteins during assembly, and pose key questions for further investigation.

## 1. Introduction

Despite being near ubiquitous in the population [1], overt human cytomegalovirus (HCMV) disease in adults is typically restricted to the immunocompromised [2,3]. Even in the era of HAART, HCMV is associated with increased risk of progression to AIDS and immune reconstitution inflammatory syndrome in HIV-infected individuals [4]. HCMV also causes significant disease and mortality in transplant recipients [3]. Such patients frequently undergo prophylactic therapy to reduce HCMV-associated complications, leading to viral resistance and development of late-onset HCMV disease [5,6]. HCMV is also the most common intrauterine infection in the U.S., with outcomes ranging from death to neurological impairment, sensorineural hearing loss, seizure disorder, cerebral palsy and chorioretinitis [7,8]. Significantly, permanent disability due to congenital HCMV infection is more common than other well-known conditions such as spina bifida and Down’s syndrome [9]. Currently available treatments for HCMV infection target the viral DNA replication machinery, and can be quite effective at diminishing the impact of HCMV disease [10]. However, strains resistant to these drugs are emerging at a rate of 5%–10% depending on the underlying condition and type of treatment [10], prompting the need to develop new antiviral strategies. The tegument proteins of HCMV have been proposed as potential therapeutic targets due to their key functions in the initiation of infection, virion assembly and particle stability [11,12]. While we know a considerable amount regarding the functions of the tegument proteins during the initial phases of virus replication [11,13], our understanding of the specific role of the HCMV tegument proteins in virus assembly lags behind. In this review, we will focus on the functions of tegument proteins in the assembly, egress and stability of virus particles.

## 2. Overview of Replication and Assembly

Replication of HCMV begins with virus binding to cell surface receptors and fusion of the viral envelope with the plasma membrane [14]. At that time, the viral capsid is released into the infected cell along with the components of the virion tegument, a layer of proteins located between the viral envelope and the capsid. The tegument consists of approximately 38 different viral proteins that are released into the host cell upon infection where they play critical roles during the initiation of virus replication [11,12]. The HCMV tegument also contains a number of cellular proteins [15] and mRNA’s [16], although it is unclear at this point if these play any role in virus infection, or if they are simply bystanders that are nonspecifically incorporated during the assembly process. During lytic replication, the tegument proteins function to enhance the efficiency of viral immediate early gene expression [11,12]. The immediate early proteins are essential for progression through the early phase of gene expression [17,18], which is followed by DNA replication, the late phase of viral gene expression [19] and virion assembly [20]. 

HCMV assembly occurs in two phases; first, the capsid is formed within the nucleus and the DNA genome is encapsidated [20]. It is likely that at this stage, the initial components of the tegument are added to form the inner tegument, an organized net-like layer that encloses the capsid shell [21]. The capsids then bud through the nuclear envelope and final assembly, including the addition of the bulk of the tegument, occurs in a unique structure consisting of redistributed components of the cellular secretory apparatus known as the assembly complex (AC) [20,22,23,24]. The majority of the tegument lacks a defined structure [21], and as mentioned above, incorporates a number of cellular proteins and mRNA’s [15,16], suggesting that final tegumentation is a relatively nonspecific process. However, there is evidence pointing to a degree of specificity for at least some of the viral tegument components. For example, while three isoforms of the pUL69 protein can be detected in infected cells, only one specific isoform of pUL69 is incorporated into the tegument [25]. Likewise, it has been reported that a hypophosphorylated form of the pp28 tegument protein (product of the *UL99* gene) is specifically incorporated into virions [26]. Another possibility to account for the difference in protein isoforms located within the virion *versus* those found in infected cells is modification within the tegument. In that regard, viral and cellular kinases are known tegument components [27,28], and the cellular phosphatases PP1 and PP2A can also be detected in the virion [29]. This raises the possibility that fine tuning of tegument protein post-translational modifications can occur within the virion and have the potential to modulate virus infection. It should be mentioned here that the majority of the tegument proteins can be phosphorylated and the abbreviation “pp” denotes this fact.

Additional studies support the notion that the HCMV tegument is added in an ordered step-wise fashion. For example, there is an abundance of evidence that addition of the pp150 tegument protein, encoded by the *UL32* gene, initiates in the nucleus [30,31], and is the primary component of the structured tegument network surrounding the capsid [21]. While the pp150 network clearly plays a role in capsid stability [32,33], it may also provide a structural framework for the addition of subsequent tegument components. Consistent with this, pp150 has been shown by mass spectrometry analysis to interact with a number of capsid and tegument proteins, including the pp71 protein encoded by the *UL82* gene [34]. This is of particular note in that pp71, along with the pp65 tegument protein encoded by the *UL83* gene, has a relatively tight association with the capsid, suggesting that it may form a secondary, albeit less organized, layer of tegument [35]. Recent studies utilizing yeast two-hybrid analysis have revealed interaction networks centered on a number of hub proteins that may contribute to an ordered tegumentation process [36,37]. For example, the pUL45 tegument protein forms a network hub via interaction with itself, as well as the pUL25, pp150, pUL48, and ppUL69 tegument proteins [36]. Although the precise function of pUL45 is unknown, deletion of this gene causes a defect in plaque formation [38]. A more extensive yeast two-hybrid analysis revealed interactions amongst a number of tegument, capsid and envelop proteins, and also identified the pUL24, pUL25 and pUL89 proteins as network hubs [37]. Intriguingly, both studies showed that a number of the tegument proteins were capable of self-association [36,37]. Such extensive networks of cross-interactions and self-interactions are likely to be critical for efficient tegumentation during the assembly process. Perhaps surprisingly, other than pUL45, most of these hub proteins are not essential for efficient virus replication [39]. A generally accepted concept that accounts for this apparent discrepancy is that multiple overlapping interactions and functions ensures that efficient tegumentation and subsequent infectivity is less dependent on any one tegument protein [20,40].

While it is clear that the majority of the tegument proteins become associated with the virion particle in cytoplasmic assembly complexes [20,40], a number of these proteins contain nuclear localization signals and transit to the nucleus at the initial stages of virus infection [11,12,41,42,43]. Thus, the tegument proteins can be considered as belonging to three major classes; the inner tegument consisting of pp150, which likely initiates tegument incorporation in the nucleus [30,31] and pUL48 [21]; nuclear tegument proteins that initially transit to the nucleus of permissive cells and are later relocalized to the cytoplasmic AC, including pp71, pp65, pTRS1, pUL26, pUL35 and the ppUL97 viral kinase [11,12,44]; and the cytoplasmic tegument proteins that retain this localization throughout the course of virus infection, including pp28, pUL71 and pUL25 [45,46,47]. One of the key questions regarding tegumentation that remains unanswered is whether the nuclear tegument proteins associate with the capsid within the nucleus. Direct evidence for the association of the nuclear tegument proteins with capsids within the nucleus is lacking, although this possibility cannot be definitively ruledout [20,40]. If the nuclear tegument proteins do not associate with capsids in the nucleus, how are they redirected to the cytoplasmic AC at the late stages of infection? For some of these proteins, including pp65, pUL94 and ppUL69, there is evidence for shuttling activity between the nucleus and the cytoplasm [48,49,50]. Thus, the relocalization of these proteins to the cytoplasm could result from modulation of the rate of nuclear import and/or export. For the pp65 protein, phosphorylation clearly plays a role in controlling this process, as inhibition of cellular cyclin dependent kinase (CDK) activity prevents the accumulation of pp65 in the cytoplasm at the late stages of virus infection [51]. In addition, the viral ppUL97 kinase affects the distribution of pp65, with inhibition of ppUL97 resulting in large pp65-containing aggregates [52,53]. However, the formation of these aggregates is more consistent with a role for ppUL97 is regulating the self-association of pp65, rather than a direct effect on trafficking, per se. With other nuclear tegument proteins, domains have been identified that can directly target the proteins to the cytoplasmic AC [43,54]. For example, in transfected cells, the pp71 tegument protein colocalizes with markers of the late-*trans* Golgi Network and early endosomal compartment if the nuclear localization signal is mutated [43]. However, the precise mechanism of this switch in trafficking during the process of virus infection has yet to be identified. Thus, significant gaps in our understanding of HCMV tegumentation remain. Despite this, continued research into the specific functions of the tegument proteins promises new insights into this process, and may yet reveal novel antiviral targets amongst this key class of viral proteins. In the remainder of this review, we will highlight the specific functions of components of the HCMV tegument during virion assembly (Table 1), with a focus on the most recent data.

**Table 1:** Tegument proteins discussed in detail in this review.

Gene Designation	Relative Abundance ^a^	Phenotype ^b^
**Inner Tegument**		
*UL32* (pp150)	9.1%	Essential [39,55]
*UL48*	12.6%	Essential [39]; Severe growth defect (>10^4^ fold drop in titer) [55]
**Nuclear Tegument**		
*UL26*	0.1%	Non-essential, Small plaque phenotype with severe growth defect [39,55]
*UL35*	0.5%	Moderate growth defect (1,000-fold decrease in titer), Essential at low multiplicity [56]
*UL82* (pp71)	8.9%	Non-essential, Severe growth defect at low multiplicity [57,58]
*UL83* (pp65)	15.4%	Non-essential [59], Important for replication in macrophages [60], Slight growth defect (10-fold) at low multiplicity in fibroblasts [61]
*UL94*	1.2%	Essential [39,62]
*UL97*	0.1%	Non-essential, Severe growth defect [63]
*TRS1*	0.6%	Non-essential, Moderate growth defect (~200-fold decrease in titer) [64]
**Cytoplasmic Tegument**		
*UL71*	0.1%	Essential [39]
*UL99* (pp28)	Unknown	Essential [39,55,65]

^a^ Data on relative abundance obtained from Varnum *et al.* [15]. Note that the relative abundance of the pUL48 protein in this report includes the entire *UL48-49* gene region. ^b^ Phenotype represents the ability of virus with a mutation in the indicated gene to replicate in fibroblasts unless otherwise stated.

## 3. The Inner Tegument Proteins

### 3.1. pp150 (ppUL32)

The product of the HCMV *UL32* gene, pp150, is the primary component of the inner tegument and forms a filamentous net-like structure that surrounds the capsid [21]. The inner tegument can be distinguished from the outer tegument proteins by virtue of this relatively organized structure, as well as its tight association with the capsid [66]. Recent studies have revealed that pp150 can be co-purified from cells at the late stage of infection in association with capsid components including the major capsid protein (MCP, product of the *UL86* gene), pUL46, pUL85, and pUL80.5 [34]. However, co‑purification of proteins at this time point probably represents a more general capsid association as opposed to a direct interaction with any one specific component. Additional studies using cryo electron microscopy (cryo-EM) reveal that the smallest capsid protein, pUL48.5, likely makes direct contact with the inner tegument [67]. These studies also determined that the inner tegument is composed of an upper and lower helical bundle joined by a long central helix, with features of the helices consistent with the predicted structure of pp150. Moreover, reduced expression of pUL48.5 mediated by ribozyme inhibition significantly reduced viral yield and resulted in the accumulation of non-infectious enveloped particles (NIEP’s) that lack viral DNA [67]. However, this latter finding is distinct from what has been observed by either deletion or knockdown of pp150 directly [32,33]. In these studies, the number of A- (empty), B- (contain scaffold proteins) and C- (DNA filled) capsids within the nuclei of infected cells are comparable in the presence or absence of pp150 [32,33]. In contrast, very few DNA containing capsids or enveloped viral particles could be identified in the cytoplasm in the absence of pp150, suggesting that this protein stabilizes DNA-containing capsids as they progress from the nucleus to the cytoplasmic AC. The discrepancy between the effects of the pUL48.5 and pp150 viral mutants may result from the approaches employed e.g., cryo-EM of extracellular particles [67] *versus* transmission EM of particles within infected cells [32,33]. However, it is also possible that pUL48.5 has additional functions in virion assembly independent of the interaction with the pp150 inner tegument protein [67]. What is clear is that the pp150 protein is critical for the efficient formation of enveloped capsids within infected cells and that the mechanism is likely related to effects on capsid stability during post-nuclear events.

Another key question is the cellular site of inner tegument addition. The preponderance of current evidence supports an initial nuclear localization of pp150 derived from the tegument [30,31], perhaps due to the tight association with the capsid [66]. Indeed, this tight association was recently shown to facilitate a novel *cis*-mode of regulation during HCMV infection [68]. In this study, tegument-derived pp150 was found to be phosphorylated by a cyclin A2-dependent mechanism resulting in restriction of HCMV replication to the G0/G1 phase of the cell cycle. Mutation of the cyclin A2-dependent phosphorylation site blocked the cell cycle restriction effects, but in a genome-specific manner, suggesting that the tight association of pp150 with at least some capsid components is retained during the initial stages of lytic replication. Newly synthesized pp150 protein appears to be both nuclear and cytoplasmic [34,69], but evidence also suggests that tegument addition begins in the nucleus, as pp150 can be found associated with nuclear B-capsids [31]. This finding would be consistent with formation of an initial inner tegument framework in the nucleus. However, it is also clear that as infection progresses, the distribution of pp150 alters to become predominantly cytoplasmic and associated with the AC [30,69,70]. Whether this reflects the accumulation of capsids within the AC during envelopment, or an independent redistribution of pp150 to facilitate completion of the inner tegument remains an unanswered question.

Another possible function for pp150 is the targeting of capsids to the site of virus assembly. Of note is that pp150 interacts with Bicaudal D1 [71], a protein involved in microtubule dependent cargo transport [72], consistent with an association with the AC [22,23,24]. Knockdown of Bicaudal D1 or inhibition of Rab6, a Bicaudal D1-interacting protein [73], resulted in decreased localization of pp150 to the AC, and reduced virus replication, although a direct effect on capsid recruitment to the AC was not examined [71,74]. Mass spectrometry analysis also identified clathrin components as pp150 interacting proteins, although a direct role for this interaction in virion assembly or localization to the AC could not be demonstrated [34].Thus the possibility that pp150 association with the capsid could participate in the recruitment of capsids to the AC still needs to be addressed experimentally. Finally, a role for the inner tegument as a scaffold that nucleates addition of the remaining tegument makes an attractive, but unproven, model. It is known that pp150 can interact with a number of tegument proteins including pUL48, pTRS1, ppUL69, ppUL97, and pp71 [34], although this may represent association with overlapping tegument interaction networks. Yeast two-hybrid analysis demonstrates direct evidence for pp150 interactions with pUL35, pUL45 and pp71 [36,37], and as already noted pp71 is thought to have a relatively tight association with capsids [35], consistent with pp150 nucleating additional tegument components. Further evidence for a role for pp150 as a tegument scaffold comes from a study examining the pUL96 tegument protein [31]. In this analysis, pUL96 was found to be added to capsids exclusively within the cytoplasm. However, mutation of pUL96 resulted in a similar defect in virus replication to that observed in the pp150 mutant viruses, with an apparent effect on the stability of capsids after or during transition from the nucleus. This data is consistent with the initial association of pp150 with capsids in the nucleus, and perhaps stabilization of the inner tegument network upon association with pUL96 in the cytoplasm, resulting in nucleocapsids capable of association with additional tegument and finally envelopment in the AC before virus egress from the cell.

### 3.2. pUL48

The protein product of the *UL48* gene, pUL48, is a large tegument protein that is tightly associated with the capsid [75], is a component of the inner tegument [21], and localizes to both the nucleus and cytoplasm of infected cells [76]. The best characterized function of the pUL48 is in viral entry where it interacts with pUL47, pUL69 and the pUL86 major capsid protein and is thought to assist in release of the viral genome from the capsid during the initial stages of infection [77]. Like its homologs in other herpesviruses, the HCMV pUL48 has deubiquitinase activity, and mutation of the active site results in a 10-fold reduction in virus yield [78,79]. However, a specific role for the deubiquitinase activity in virus assembly has not yet been shown. In a more recent study, the nuclear localization signal of pUL48 was mapped, and disruption of nuclear trafficking resulted in a small plaque phenotype [76]. While the nuclear localization could clearly be involved in the established role for pUL48 in virus entry, it also raises an intriguing possibility that this localization could contribute to the initial stages of tegumentation along with pp150. The pUL48 protein has also been shown to interact with the cellular p180 ER membrane-associated protein [80], and thus could play a role in directing capsids to the cytoplasmic AC. Consistent with this, deletion of the herpes simplex virus (HSV) homolog (pUL36 or VP1/2) results in the accumulation of cytoplasmic unenveloped capsids [81]. Detailed analysis of the HSV pUL36 protein during assembly shows that the protein is added to cytosolic capsids and functions to link the inner and outer tegument [82,83]. It remains to be seen whether the HCMV pUL48 protein functions in a similar manner.

## 4. The Nuclear Tegument Proteins

It has been well characterized that a number of HCMV tegument proteins, including pp71 and pp65 (products of the *UL82* and *UL83* genes, respectively), initially localize to the nucleus of permissively infected cells [11,12,84,85]. Of note, both pp71 and pp65 contain nuclear localization signals that can independently target the proteins to the nucleus [41,43]. The nuclear trafficking of these proteins during the initial stages of virus infection is associated with their role in enhancing the efficiency of replication and viral gene expression [11]. For example, the pp71 protein has been shown to enhance the transcription of the major immediate early gene region via degradation or relocalization of transcriptional repressors such as Daxx and ATRX [13]. In a similar vein, the pp65 protein was recently shown to enhance activation of the major immediate early promoter via its interaction with the IFI16 protein [61]. It has also been observed that these initially nuclear tegument proteins relocalized to the AC at the late stages of virus replication. While the precise mechanisms of redistribution remain unclear, as noted above phosphorylation does appear to play a role in some cases [43,51,53]. What is perhaps surprising is that a number of these tegument proteins, including pp65 and pp71, are not essential for virus replication [12], emphasizing the apparent redundancy in tegument protein function [20,40]. Despite this, some of the nuclear tegument proteins have been shown to play important roles in virion assembly and stability.

### 4.1. pUL26

The pUL26 protein follows the standard pattern of nuclear tegument protein localization, with an initial nuclear distribution, followed by a perinuclear localization consistent with the AC [44]. When infections with a virus lacking pUL26 were carried out at low multiplicity, expression of the viral immediate early protein levels were reduced relative to the wild type virus, leading to a corresponding decrease in the efficiency of viral DNA replication. Of particular note with respect to assembly, this study showed that the levels of the pp28 and pp65 tegument proteins were diminished within the newly infected cells, although virion levels of both proteins were similar to wild type virus [44]. This finding suggested that the proteins in the mutant virus were somehow less stable, and may correspond to an altered hypophosphorylated form of pp28 being preferentially incorporated into the particles. In a separate study of a pUL26 deletion virus, the authors noted a decrease in particle stability [86]. Specifically, the wild type and revertant viruses were relatively stable (~50% infectivity) for at least two days at 20 degrees, whereas the pUL26 deletion virus retained only 5% infectivity over the same time period. Electron microscopy revealed nonenveloped extracellular particles derived from cells infected with the pUL26 deletion virus, suggesting that the decrease in stability resulted from a loss of the viral envelope due to defective tegumentation. Such an interpretation would be consistent with a role for the tegument analogous to the matrix proteins of RNA viruses in which the tegument forms a critical link between the capsid and the viral envelope [87].

The absence of pUL26 also resulted in the accumulation of immature particles in the cytoplasm associated with electron dense areas, possibly representing aggregates of tegument proteins [86]. This finding is consistent with other data suggesting that disruption of tegumentation leads to nonspecific aggregation of the tegument proteins [53,56], and implies that the interactions amongst the tegument proteins must be tightly regulated. The deletion of the *UL26* gene also resulted in increased levels of pp71 in the virion particles [86]. As there is no direct evidence for interaction between pUL26 and pp71, the authors speculate that pUL26 and pp71 may compete for binding to the same capsid protein, and without pUL26, more pp71 becomes incorporated into the virion, perhaps as a compensatory mechanism. This may be one mechanism that accounts for the apparent redundancy of a number of the tegument proteins [20,40].

### 4.2. pUL35

The *UL35* gene codes for two isoforms that differ in size; the full-length protein that is expressed at the early stages of infection, and pUL35a consisting of the carboxy-terminal region that is expressed with late kinetics [88]. Both isoforms can interact with the pp71 tegument protein, although there are conflicting reports as to the effect of this interaction on viral gene expression [88,89]. Recent studies suggest a role for both pUL35 and pUL35a in controlling ND10 reorganization [90], known to be important for viral replication and gene expression [91]. In addition, pUL35 can interact with and regulate the cellular DNA repair machinery [92]. Deletion of the *UL35* gene from HCMV results in decreased numbers of both enveloped viral particles and dense bodies (subviral particles consisting primarily of tegument surrounded by the viral envelope [93]) in the cytoplasm under conditions where viral gene expression is unaffected, suggesting an additional defect in virus assembly [56]. The mechanism of the defect is likely related to the persistence of the pp65 and pp71 proteins in the nucleus, where they form large electron dense accumulations [56], suggesting that the lack of *UL35* proteins disrupts the relocalization of these proteins to the AC. Further analysis showed that the smaller isoform, pUL35a, was largely responsible for this effect, as co-transfection of pUL35a with pp71 results in relocalization of both proteins from the nucleus to the cytoplasm [90]. Interestingly, the two proteins colocalized in the cytoplasm in distinct punctate perinuclear structures, similar to those observed when pp71 nuclear localization is abrogated by a specific mutation within the pp71 nuclear localization signal [43]. Together, these findings suggest that pUL35a disrupts recognition of the pp71 non-classical NLS, either through a conformational change or altered phosphorylation, resulting in the nascent pp71 AC localization predominating [43]. It is less clear how deletion of the *UL35* gene alters pp65 redistribution, as there was no evidence of a direct interaction between these two proteins in the yeast two-hybrid assays [36,37]. However, recent studies in our laboratory show that affinity purification of the pp71 protein from cells at the late stages of infection results in the co-purification of a number of viral proteins, including pp65 (Table 2). These findings suggest that pp71 and pp65 may exist as a complex, and pUL35a could redistribute both proteins to the cytoplasm at the later stages of virus infection [88]. Further, the phenotype of the *UL35* gene deletion virus is consistent with sustained retention of the nuclear tegument proteins within the nucleus decreasing the efficiency of virion morphogenesis in the cytoplasm.

**Table 2:** Viral proteins that co-purify with pp71 ^a^.

Protein Name	Protein Function	# of Peptides Matched	Ion Score ^b^ (−10Log(P))	Sequence Coverage
pTRS1	Transcriptional Regulation/Immune Evasion [12]	78	2439	44%
pIRS1	Transcriptional Regulation/Immune Evasion [12]	60	1809	41%
pUL44	DNA Processivity Factor [94]	66	2413	82%
pUL86	Major Capsid Protein [95]	5	339	8%
ppUL97	Viral Kinase [96]	5	272	8%
pp65	Tegument protein [12]	236	2580	71%
pUL50	Nuclear Egress [97]	9	276	26%
pUL52	Genome Cleavage and Packaging [98]	7	256	14%
pUL56	Terminase Subunit [99,100]	11	385	26%
pUL88	Tegument Protein [100]	7	135	10%
pUL35	Tegument Protein [12]	6	289	29%

^a^ Proteins were purified using S-protein agarose (Novagen) from cells infected at 72 hpi with a virus expressing a S-tagged version of pp71 [101]. The resultant proteins were subjected to SDS-PAGE using a 4%–20% gel (Jule Biotechnologies, Inc., Milford, CT, USA). Protein bands were visualized by Coomassie Blue staining, excised and subjected to trypsin digestion prior to analysis on an LTQ^TM^ Linear Ion Trap tandem Mass Spectrometer (ThermoFinnigan, San Jose, CA, USA). Protein searches were performed using MASCOT^TM^. Only proteins identified in multiple experiments are included. ^b^ The ion score is based on the calculated probability, P, and provides a measure of the likelihood that the predicted peptide matches the indicated protein (Matrix Science, Boston, MA, USA). For the study shown, a score of >36 indicated identity or extensive homology (*p* < 0.05).

### 4.3. pp71 (ppUL82)

The pp71 tegument protein, encoded by the HCMV *UL82* gene, plays a number of roles during the initiation of virus replication, including the degradation and/or inactivation of cellular proteins that would repress viral gene expression [13]. The majority of research on pp71 has focused on its functions during the establishment of infection, and it is unclear what role if any it may play during virus assembly. However, some studies are suggestive of a contribution. For example, as noted above delayed relocalization of the pp71 protein, along with pp65, to the cytoplasm due to the deletion of the *UL35* gene results in a decrease in the efficiency of secondary envelopment [56]. A similar effect was observed upon deletion of the *TRS1* gene [64]. Other evidence that points to a role in virion assembly is the possibility that pp71 interacts with a number of key virion components including pp150, pUL94, pUL35, pIRS1/TRS1 and the major capsid protein (Table 2) [36,37]. Despite these findings, a definitive role for pp71 in assembly remains to be demonstrated.

However, analysis of pp71 trafficking and post-translational modifications have revealed insight into the mechanisms involved in nuclear tegument protein trafficking and redistribution [43]. In this study, a large region from amino acids 94–300 termed the mid-region (MR) of pp71 was found to be necessary and sufficient for nuclear localization, suggesting the presence of a large non-classical nuclear localization signal. Phospho-mapping of pp71 from transfected cells revealed a single phosphorylation site within the MR, at threonine 223 (T223). Mutation of T223 to a phosphomimetic resulted in a block in nuclear localization, and redistribution of pp71 in a punctate perinuclear region. Confocal analysis using intracellular markers demonstrated that cytoplasmic pp71 colocalized with markers of the late *trans*-Golgi Network and late endosomal compartments. Notably, these cellular components are reorganized along with other structural elements of the secretory pathway into the cytoplasmic assembly complex in infected cells [22,23,24]. Together, these findings are consistent with pp71 containing an integral signal that denotes trafficking to the AC that is regulated by phosphorylation [43]. Ongoing studies in our laboratory are directed towards determining the significance of these findings during virus infection.

### 4.4. pp65 (ppUL83)

The pp65 protein is the most abundant component of the tegument, and like the other nuclear tegument proteins initially traffics to the nucleus of permissively infected cells, and then relocalizes to the cytoplasm at the later stages of infection [42,70]. While initial reports showed that pp65 played no role in virus infection *in vitro* [59], more recent studies show that pp65 is important for efficient growth in both monocyte-derived macrophages and fibroblasts [60,61,102]. The underlying cause of this growth defect likely involves pp65 functions during the initiation of infection as well as during assembly. For example, pp65 can enhance the activation of the major immediate early promoter through its interaction with the cellular IFI16 transcriptional regulator [61]. Disruption of pp65 expression can also influence the incorporation of other proteins into the virion tegument, such as pUL25, ppUL97 and ppUL69, suggesting that pp65 forms part of a protein interaction network that may be important for assembly [60]. This finding is consistent with yeast two-hybrid analysis that identified pUL25 as a hub protein for an interaction network that included pp65 [37], and studies demonstrating a direct interaction between ppUL97 and pp65 [103]. In spite of these changes to the tegument composition, the virion particles appeared indistinguishable from those resulting from the wild type virus, except for the absence of dense bodies. In this study, a defect in replication of the pp65 mutant virus in monocyte-derived macrophages was observed, although whether this is due to the absence of pp65 per se, or the lack of important viral proteins such as ppUL97 [63] and ppUL69 [104] during the initial stages of replication remains to be definitively established.

A virus containing a 30 amino acid insertion within the pp65 open reading frame, termed RV-VM1, also displayed a defective growth phenotype [102]. In this case, the defect was most likely caused by the retention of pp65 in the nucleus at the late stages of infection, resulting in accumulation of the MCP in the nucleus, reduced numbers of C-capsids in the cytoplasm and a lack of dense body formation. The lack of pp65 relocalization resulted in the formation of large globular structures within the nucleus that contained the pp65 and ppUL69 proteins. Surprisingly, the redistribution of pp150 and pp71 to the AC were unaffected by the mutation, suggesting that these proteins trafficked independently of pp65 in this case. One possible explanation for this effect is that the insertion within pp65 disrupts the interaction with the pp71 protein. The movement of pp150 into the AC despite the retention of immature capsids in the nucleus is harder to explain, but may be consistent with an independent redistribution of pp150 to the AC to facilitate completion of the inner tegument. The lack of C-capsids in the cytoplasm of cells infected with RV-VM1 implies a direct role for pp65 and/or ppUL69 in capsid egress from the nucleus. However, the possibility that nonspecific effects due to the formation of the large globular structures within the nucleus also play a role in this phenotype cannot be excluded. Interestingly, inhibition of ppUL97 kinase activity also results in the accumulation of pp65 in the nucleus of infected cells where it forms large nuclear inclusions, presumably due to self-interaction [53].

**Figure 1:**
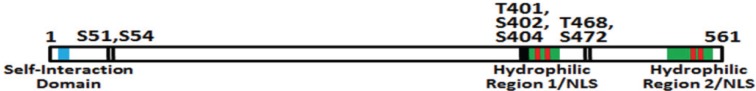
Schematic of pp65 phosphorylation sites at the late stages of virus infection. The pp65 protein was purified from cells infected at 72 hpi by virtue of its association with the pp71 protein (See Table 2). The band corresponding to pp65 was excised from an SDS‑PAGE and subjected to mass spectrometric analysis as previously described [43]. Also shown are the pp65 hydrophilic regions (green), sequences required for nuclear localization (red) and the self-interaction domain (blue) [41,59,106].

Cumulative evidence points to the regulation of pp65 localization and/or self-interaction as important for efficient virion assembly and egress. In that regard, pp65 can shuttle in and out of nucleus with export occurring in a CRM1 dependent manner [48,51]. As mentioned above, control of the rates of nuclear import and export would provide a useful mechanism for the regulation of pp65 localization. Interestingly, the pp65 interacting protein, ppUL69 also functions as a shuttling protein, although it is thought that this protein functions primarily to transport mRNA’s from the nucleus to the cytoplasm [105]. Other tegument proteins also regulate the relocalization of pp65, including pUL35, pTRS1 and pUL96 [31,56,64]. It is likely that phosphorylation of pp65 also plays a role in regulating the localization of this protein during infection. For example, the ppUL97 kinase is clearly involved in the regulation of pp65 redistribution to the AC at the later stages of infection [53] and pp65 is a direct target of the ppUL97 kinase [102]. Infection in the presence of CDK inhibitors also affects both the phosphorylation and localization of pp65 [51]. In order to examine the effect of phosphorylation on pp65 localization and function, we recently used mass spectrometry to determine the sites on which pp65 was phosphorylated at the late stages of infection (Figure 1). This analysis revealed clusters of phosphorylation sites flanking the pp65 nuclear localization signals [41,42], suggesting that phosphorylation may influence the recognition of these signals and alter the distribution of pp65. Interestingly, the 30 amino acid insertion in the RV-VM1 virus occurs at amino acid 387 [102], also in relatively close proximity to the pp65 nuclear localization signals. Indeed, this mutation reduces the susceptibility of the mutant virus to ppUL97 kinase inhibitors. One model to explain this data is that phosphorylation of pp65 by ppUL97 blocks recognition of the nuclear localization signals, resulting in a change in distribution to the cytoplasm. The insertion in the RV-VM1 virus could result in a different pattern of phosphorylation that results in an active nuclear localization signal and subsequent accumulation and aggregation of the protein in the nucleus. Interestingly, two phosphorylation sites were also clustered in the amino terminus in proximity to the self-interaction domain, suggesting that phosphorylation also regulates this property of pp65. This data is certainly consistent with the large aggregates of pp65 that form in the absence of ppUL97 activity [53].

### 4.5. pUL94

The product of the *UL94* gene is nominally within the group of nuclear tegument proteins as it has been shown to be located in the nucleus of both transfected and infected cells [62,107]. Indeed, similar to the pp65 protein, pUL94 has nuclear:cytoplasmic shuttling activity [50]. However, the major functions of pUL94 during infection that have been reported are associated with its ability to interact with pp28, a cytoplasmic tegument protein [62]. Deletion of the *UL94* gene results in defective replication, although viral gene expression and DNA replication are unaffected. The defect in replication is attributed to the accumulation of non-enveloped capsids in the cytoplasm, a phenotype similar to that observed upon deletion of the *UL99* gene that encodes pp28 [65]. Thus, it has been concluded that pUL94 works with pp28 to facilitate secondary envelopment of tegumented capsids in the cytoplasm. It has been proposed that pUL94 may regulate the trafficking of the pp28 protein to the cytoplasmic AC [62,108]. This finding is in contrast to earlier analysis of the pp28 protein in the absence of virus infection, where it was found to localize to the endoplasmic reticulum-Golgi intermediate compartment, suggesting that trafficking to membranes associated with the AC may be an integral function of the pp28 protein [109]. Despite this discrepancy, it is clear that pUL94 and pp28 work in concert to facilitate secondary envelopment of HCMV virions.

### 4.6. ppUL97

The ppUL97 viral kinase was initially identified by virtue of its ability to phosphorylate the antiviral compound ganciclovir [110]. The kinase activity of the ppUL97 protein is critical for efficient virus replication and it has therefore been the subject of intense investigation as an antiviral target [111]. In regards to virion assembly, a notable function of the ppUL97 viral kinase is in the redistribution of nuclear tegument proteins to the AC, as previously mentioned [53,102]. In the absence of this relocalization, the tegument proteins form large nuclear aggregates, suggesting that ppUL97 may regulate the self-association of these tegument proteins [111]. In addition, as the ppUL97 protein can be incorporated into virion particles [28], it is possible that this kinase may modulate phosphorylation of virion components *in situ*, or immediately after infection. Recent studies also show that ppUL97 can interact with pUL50, and together with pUL53 disrupts the nuclear lamina, enabling efficient capsid egress from the nucleus [112,113]. The ppUL97 protein has also been proposed to play a direct role in the formation of the cytoplasmic AC [52,114]. Specifically, deletion of the HCMV *UL97* gene or the use of ppUL97 kinase inhibitors results in a modified AC which is more diffuse than that observed in a typical infection, together with the formation of large perinuclear vacuoles [114]. Immuno-electron microscopy revealed that these cytoplasmic vacuoles were surrounded by membranes and contained accumulations of tegument proteins, including pp28 and pp65, as well as the gB envelope protein. The similarity of these vesicles to the nuclear inclusions observed upon inhibition of ppUL97 [111] add further weight to the contention that self-association of tegument proteins is tightly regulated within infected cells. Interestingly, a more severe growth defect was observed when the UL97 gene was deleted *versus* a kinase dead mutant, or in the presence of a ppUL97 kinase inhibitor [52]. Of note, the presence of dense bodies, tegumented cytoplasmic capsids and viral particle release were detected in the presence of the kinase dead mutant or when the kinase inhibitor NGIC-I was added to the infected cells. However, when the *UL97* gene was deleted, dense bodies were absent, as was evidence of particle release, and only a few C-capsids were observed in the cytoplasm. This finding suggests that the ppUL97 protein has non-kinase related functions in virion assembly and formation of the AC. However, the possibility that kinase inhibition was not 100% efficient in the ppUL97 mutant or in the presence of the inhibitor cannot be ruled out. Regardless, further identification of cellular and viral targets of ppUL97 will likely yield more insights into the precise role of this enzyme in virion assembly and egress.

### 4.7. pTRS1

The *IRS1* and *TRS1* genes located in the viral repeat sequences code for highly related, although not identical proteins [115]. The products of both genes are involved in immune evasion through inactivation of the interferon induced kinase, PKR [12]. Uniquely, the pTRS1 protein appears to also be involved in virion assembly. Deletion of the *TRS1* gene results in the formation of abnormal viral particles containing decreased levels of the pp65 and pp150 tegument proteins [64]. Extracellular virus particles generated in the absence of pTRS1 also displayed abnormal sedimentation in glycerol-tartrate gradients, indicative of defective particles, with an increase in the formation of dense bodies. A delay in the movement of tegument proteins such as pp65 and pp71 into the cytoplasm at the late stages of infection was also observed, although the large nuclear aggregates observed when ppUL97 is inhibited were not noted. Further analysis of the *TRS1*-deletion virus revealed decreased capsid formation in the nucleus, suggesting that pTRS1 influences events relatively early in the assembly process [116]. However, the precise function of pTRS1 in capsid assembly remains to be determined. One possibility is that the regulation of the interferon induced kinase, protein kinase R or PKR, by pTRS1 may be involved [117]. This could result in altered phosphorylation of the tegument proteins, either through the direct inhibition of PKR activity, or altered access to substrates by virtue of the relocalization of PKR to the nucleus of infected cells [118]. Some intriguing hints have come from recent studies examining a multiprotein complex that includes pUL84, the ppUL44 DNA processivity factor and the pIRS1/pTRS1 proteins [119,120]. Deletion of the amino-terminus of pUL84 diminishes the formation of this complex, and results in an unusual distribution of nuclear capsids in the nucleolar region, as well as a lack of cytoplasmic capsids. This finding suggests that pTRS1 may participate in a complex that regulates the maturation and egress of HCMV capsids.

## 5. The Cytoplasmic Tegument Proteins

### 5.1. pUL71

Two independent studies have implicated a role for the pUL71 protein in secondary envelopment [46,121]. Specifically, deletion of the *UL71* gene results in an accumulation of non-enveloped capsids in the AC region of infected cells. In addition, enlarged vesicular structures reminiscent of multi-vesicular bodies were noted, along with distinct changes in the AC including the redistribution of cellular AC markers. At this point, it is unclear whether these observations represent the result of direct effects of pUL71 on the formation of the AC, or whether these changes are a secondary consequence of the defect in secondary envelopment. While the precise role of pUL71 in envelopment has yet to be determined, recent data shows that the oligomerization of pUL71 into dimers, trimers and higher-ordered multimers are important for this process [122]. Surprisingly, the pUL71 protein was also found to interact with pUL51 and pUL89 proteins using yeast two-hybrid analysis [37]. The pUL51 and pUL89 proteins are two components of the three subunit terminase subunits required for the cleavage and packaging of DNA within nuclear replication compartments [123]. This finding is inconsistent with the phenotype of the *UL71* deletion viruses [46,121], and thus may represent an aberrant result due to the artificial nature of the yeast two-hybrid assays.

### 5.2. pp28

The product of the *UL99* gene, pp28, localizes to the cytoplasmic AC in association with cellular membranes as a result of myristoylation in the amino terminus of the protein [45,70]. Deletion of the *UL99* gene revealed that it is essential for virus replication, and results in the accumulation of tegumented capsids in the cytoplasm, suggesting that the pp28 protein plays a critical role in secondary envelopment [65,124]. Detailed mutational analyses of the pp28 open reading frame have identified distinct domains required for both AC localization and virion assembly with an acidic cluster within the amino terminus being critical for the assembly functions [26,109]. Surprisingly, the first 50 of the 190 amino acids of pp28 was capable of conferring both AC localization and at least partially restored the defect in virion assembly [109,125]. Importantly, the incorporation of other tegument proteins into the virion particles was unaffected, suggesting that pp28 functions after the addition of the bulk of the virion tegument [125]. Such a role is consistent with the membrane association of the pp28 protein, and indeed multimerization of the pp28 protein has been proposed to promote membrane deformation and virion budding, akin to the matrix proteins of other viruses [126]. The multimerization function also maps to the first 50 amino acids of pp28, as does its ability to interact with the pUL94 protein that functions with pp28 to facilitate secondary envelopment [108].

It is somewhat surprising that these key functions of the pp28 protein are localized to such a small region with the amino terminus of the protein. One possibility is that additional non-essential functions reside within the remaining portion of the protein. In that regard, recent analysis using mass spectrometry have revealed potential interactions between pp28 and additional viral proteins, including pp65, the major capsid protein and the pUL44 DNA processivity factor [34]. These interactions may represent a component of the functional redundancy of some of the tegument proteins as previously noted [20,40]. However, it remains to be seen whether the carboxy-terminal region of pp28 participates in these interactions, or has additional hitherto unknown functions. Another unanswered question in relation to pp28 is the role of phosphorylation in any or all of its functions. It was originally reported that only a hypophosphorylated form of pp28 was packaged into virions [26]. In contrast, deletion of the *UL26* gene causes a unique hypophosphorylated form of pp28 to be incorporated into virions and this corresponded to decreased stability of the pp28 protein upon infection [44]. The reason for this apparent discrepancy is unclear, but could be related to the differences in methodologies used for the analysis. The original observation was made using mobility in standard SDS-PAGE analysis [26] whereas the second study utilized 2-D gel electrophoresis but did not compare the results to the proteins found within the infected cell [44]. Regardless, it is clear that phosphorylation of pp28 could have a major impact on its function during assembly.

## 6. Conclusions

Insights into the roles of HCMV tegument proteins during virion assembly have advanced in recent years due to enhancements in technology, most notably the ability to rapidly generate viral mutants, and more widespread use of sophisticated microscopy techniques. We now have a more complete understanding of the inner tegument components and how they interact with and stabilize the viral capsid. In addition, we are beginning to decipher the networks that link the nuclear tegument proteins and presumably facilitate their incorporation into the virion particle. We also have important clues regarding the role of the cytoplasmic tegument proteins in secondary envelopment. However, there is still much to be learned and basic questions regarding the mechanism of tegumentation remain unanswered. Specific questions include the potential role of viral and host enzymes in the regulation of virion components *in situ*; how the addition of the nuclear tegument proteins is coordinated, including the mechanism of redistribution during infection and the role of the capsid in this process; the role of tegument in targeting capsids to the assembly complex; how self-association of the tegument proteins is regulated; identification of specific cellular and viral targets of the ppUL97 viral kinase; and the nature of tegument protein redundancy. Answering these questions are not only vital to our overall understanding of HCMV assembly and egress, but are critical to realize the potential of the tegument proteins as therapeutic targets for intervention.
